# Role of Nanocomposite Support Stiffness on TFC Membrane Water Permeance

**DOI:** 10.3390/membranes8040111

**Published:** 2018-11-18

**Authors:** Jaime A. Idarraga-Mora, Anthony S. Childress, Parker S. Friedel, David A. Ladner, Apparao M. Rao, Scott M. Husson

**Affiliations:** 1 Department of Chemical and Biomolecular Engineering, Clemson University, 127 Earle Hall, Clemson, SC 29634, USA; jidarra@g.clemson.edu (J.A.I.-M.); psfried@g.clemson.edu (P.S.F.); 2 Department of Physics and Astronomy, and Clemson Nanomaterials Institute, Clemson University, Clemson, SC 29634, USA; childre@g.clemson.edu (A.S.C.); arao@clemson.edu (A.M.R.); 3 Department of Environmental Engineering and Earth Sciences, Clemson University, 342 Computer Court, Anderson, SC 29625, USA; ladner@clemson.edu

**Keywords:** thin-film composite, stiffness, water permeance, support layer

## Abstract

This paper discusses the role played by the mechanical stiffness of porous nanocomposite supports on thin-film composite (TFC) membrane water permeance. Helically coiled and multiwall carbon nanotubes (CNTs) were studied as additives in the nanocomposite supports. Mechanical stiffness was evaluated using tensile tests and penetration tests. While a low loading of CNTs caused macrovoids that decreased the structural integrity, adding higher loads of CNTs compensated for this effect, and this resulted in a net increase in structural stiffness. It was found that the Young’s modulus of the nanocomposite supports increased by 30% upon addition of CNTs at 2 wt %. Results were similar for both types of CNTs. An empirical model for porous composite materials described the Young’s modulus results. The nanocomposite supports were subsequently used to create TFC membranes. TFC membranes with stiffer supports were more effective at preventing declines in water permeance during compression. These findings support the idea that increasing the mechanical stiffness of TFC membrane nanocomposite supports is an effective strategy for enhancing water production in desalination operations.

## 1. Introduction

**Figure 1:**
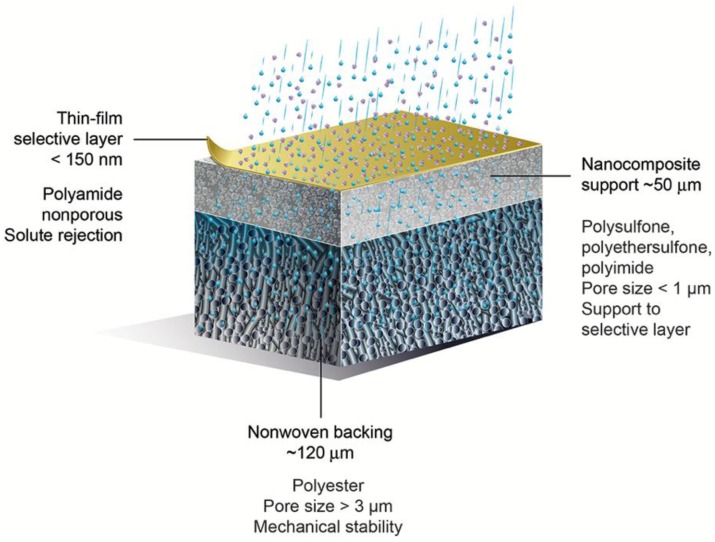
Schematic of a thin-film composite (TFC) membrane, and typical characteristics of its layers.

Seawater desalination and water reclamation via reverse osmosis (RO) are processes with high specific energy consumption [1,2]. RO energy costs can be reduced by combining this unit operation with other membrane technologies, such as pressure-retarded osmosis (PRO) or forward osmosis (FO) [1,3,4,5]. The removal of larger molecules (compared to the salt in seawater) from water is obtained at lower energy expense via nanofiltration (NF) [6]. Thin-film composite (TFC) membranes are used widely for RO and NF, and they have been studied for PRO and FO applications. Figure 1 shows a schematic representation of a TFC membrane and common values for thickness, pore size, and the chemistry of each component layer.

The performance of TFC membranes is commonly evaluated by measuring transport properties such as water permeance (*A*), salt reverse flux coefficient (*B*), and the structural parameter (*S*) [7,8]. Examples of strategies to improve these properties are surface modifications (e.g. patterning and coating), structure-controlled fabrication (e.g. laser etching and slow coagulation), and the use of additives [9]. The latter involves the incorporation of organic and/or inorganic materials, either in a liquid or solid state, to form a mixed matrix or composite structure that benefits from the properties of both materials [10].

The incorporation of carbon nanomaterials has been investigated to improve TFC membrane performance [11]. Alberto et al. [12] fabricated thin films using PIM-1 and functionalized graphene oxide nanosheets to create a separation layer on TFC membranes for n-butanol recovery from water via pervaporation. They found that adding 0.05% of graphene oxide increased the water flux through the membrane. Lai et al. [13] deposited an interlayer of graphene oxide nanosheets between the polyamide and support layers of a TFC nanofiltration membrane. They showed an enhancement of 31.4% in the water permeance of the membrane, which was attributed to the increased hydrophilicity of the membrane. Lee et al. [14] used thickness-controlled graphene oxide and polysulfone (PSf) to fabricate composite support layers for TFC membranes. The authors showed that the mechanical properties of the support increased up to 1.0% of carbon loading, and then decreased at higher loading, due to the facile agglomeration of graphene oxide sheets and the high porosity of the PSf supports. 

More specifically, for the case of multi-wall carbon nanotubes (MWCNTs), Zhao et al. [15] showed that increased loading of MWCNTs in the active layer of a TFC RO membrane modified its structure and led to higher water flux with minimal decreases in the rejection of sodium chloride. Son et al. [16] chemically functionalized carbon nanotubes and incorporated them into the support layer of a TFC membrane for desalination. This approach led to increased hydrophilicity and improved organic fouling resistance of the TFC membrane, due to the positive charge of the composite structure. Later, they showed that CNT-induced porosity also played a role in increasing water flux through the membrane [17]. Kim et al. [18] incorporated MWCNTs up to 5.0 wt % in the support layer of a TFC membrane, yielding enhancements of up to 20% in pure water permeability. The enhancements were attributed to the hydrophilicity of the modified MWCNTs and the selective flow through the MWCNT nanopores. Song et al. [19] conducted FO experiments with double-skinned TFC membranes loaded with MWCNTs in the active layers. These membranes displayed a higher water flux than membranes without MWCNTs. Additionally, MWCNT-loaded membranes showed a higher recovery flux after three cycles of fouling and cleaning.

More recently, Lee et al. [20] fabricated MWCNT–polyaniline complexes and introduced them into a polyethersulfone (PES) ultrafiltration membrane to remove natural organic matter (NOM) from the water. Addition of these complexes increased porosity, narrowed pore size distribution, increased hydrophilicity, and introduced a positive charge to the PES membrane, factors that resulted in higher water permeance and a fourfold higher NOM rejection (80%) than pristine PES membranes. Finally, Shawky et al. [21] performed measurements of mechanical properties of a composite membrane made with MWCNTs grafted onto the polyamide selective layer, obtained by reacting m-phenylenediamine and isophthaloyl chloride. They found proportional increases of the Young’s modulus and tensile strength of the membranes with the addition of MWCNTs. In addition, the MWCNT incorporation increased the hydrophobicity and both the sodium chloride and organic matter rejection of the membrane.

In all of these reports, incorporation of MWCNTs led to improved water permeance without compromising rejection. However, this effect has been attributed in many cases to changes in chemistry, even though similar results have been obtained with opposite trends in hydrophilicity. We espouse the view of Wang et al. [22] that membrane mechanical properties also influence membrane performance. The mechanical properties of MWCNTs and their behavior in polymer composites have been investigated over the last three decades [23,24,25,26]. Also, a relatively recent development is the creation of helically coiled carbon nanotubes (HCNTs), and there has been much less inquiry into the properties of these materials when used in nanocomposites. Vertically aligned arrays of HCNTs and MWCNTs have both been shown to have excellent compressive properties [27,28], which may be important for pressure-driven membrane applications. Wang et al. [22] reviewed the importance of knowing mechanical properties in order to estimate the failure mechanisms and loss of dimensional stability of the membranes. They presented the most widely used mechanical characterization techniques in membrane science, and discussed how mechanical properties relate to membrane performance.

Based on the overall literature review, we hypothesize that changes in the composite structure and stiffness due to CNT incorporation play a vital role in the improved performance in TFC membranes with these additives. The overall structure of a composite support is related intrinsically to its mechanical behavior, but data on the mechanical properties of nanocomposite membrane supports that include CNTs usually are not presented. Additionally, comments on the orientation or shape of the CNT additives, and comparisons with other types of CNTs are rarely reported. No data have been presented when using HCNT membrane composites.

To address these knowledge gaps, a study was done to understand the role(s) of the addition of CNTs (MWCNTs and HCNTs) in the structural stiffness of porous polymer films prepared via wet phase inversion and used as supports for the fabrication of TFC membranes. Mechanical stiffness was evaluated using tensile tests (Young’s modulus) and penetration tests. A model was developed and used to analyze the Young’s modulus of our porous nanocomposite membranes and other composite membranes. The effect of the mechanical stiffness of the nanocomposite support on TFC membrane water permeance under compression was studied.

## 2. Experimental

### 2.1. Materials

Matrimid® 5218 US (from here Matrimid) was kindly provided by the Huntsman University Program (Huntsman Corporation, The Woodlands, TX, USA). Ferrocene (98%), hydrogen peroxide (30 wt % in water), m-phenylenediamine (MPD, flakes, 99%), n-hexane (anhydrous), N-methyl-2-pyrrolididone (NMP, ACS reagent, 99%), nitric acid (ACS reagent >90%), poly(ethylene glycol) Bio-Ultra 400 (PEG 400), o-xylene (reagent grade), sodium chloride (NaCl, BioXtra, >99.5% (AT)), sodium hypochlorite solution (NaClO_(aq)_, reagent grade, available chlorine 10–15%), and trimesoyl chloride (TMC, 98%) were purchased from Sigma-Aldrich Corporation (St. Louis, MO, USA). Indium isopropoxide (99.9%) and tin isopropoxide (99%) were obtained from Alfa Aesar (Haverhill, MA, USA). Sodium bisulfite (NaHSO_3_, reagent grade, granular) was purchased from Fisher Science Education (Thermo Fisher Scientific, Waltham, MA). Deionized (DI, 18 MΩ⋅cm) water was prepared using a Milli-Q water purification system from Millipore-Sigma (Billerica, MA, USA). MWCNTs with nominal dimensions of 50 nm diameter and 20 µm length were purchased from Cheap Tubes Inc. (Grafton, VT, USA).

CNT synthesis and dispersion details are given in Supporting Information. MWCNTs and HCNTs samples underwent the same treatment before final dispersion in NMP. The nanotubes were sonicated in 3 M nitric acid for 30 min followed by boiling for 2 h while stirring. Boiling was carried out in a hood with adequate ventilation and personal protective equipment. This process was then repeated with 30% hydrogen peroxide solution before the nanotubes were filtered and rinsed thoroughly with water. They were dried before dispersing in NMP.

### 2.2. Fabrication of TFC Membranes with Nanocomposite Supports

#### 2.2.1. Casting Nanocomposite Supports with Carbon Nanotubes and Matrimid

For casting nanocomposite supports, dope solutions were prepared using a constant formulation of 18% polymer, 16% PEG 400, and 66% NMP (all in weight percent). This formulation was based on previous studies using Matrimid as described by Han et al. [29]. CNTs were added as a dispersion in NMP. Han et al. also showed that Matrimid, under proper conditions, can form fully sponge-like cross-sectional structures. On the other hand, when Hoek and coworkers [30] investigated the use of nanocomposites made with several nanoparticles and PSf, scanning electron microscopy (SEM) imaging of the cross-sections showed that PSf supports tend to form macrovoids. Therefore, we chose Matrimid for its sponge-like structure, with the goal being to increase the contact between the polymer and CNTs, and to improve the mechanical stress transfer [31]. The masses of pure NMP and CNT dispersion were adjusted to keep the NMP content at 66 wt%. The load of CNTs ranged from 0 to about 2 wt % with respect to the Matrimid (i.e., in the polymer matrix). All materials were placed in 250 mL Erlenmeyer flasks, sealed with stoppers and parafilm, and heated in a glycerol bath at 70 °C with magnetic stirring at ~100 rpm overnight. After mixing, the stirrer was recovered and the solution was left in the bath until air bubbles were not visible. The dope solutions were then cooled to ambient temperature. 

Nanocomposite supports were produced by phase inversion in a non-solvent bath (DI water). For samples prepared at low humidity, a home-built glovebox with a nitrogen purge was used to reduce the relative humidity of the environment in contact with the polymer solution below 15%, measured with a humidity indicator (Extech 445814, Extech Instruments, Waltham, MA, USA). A film of dope solution was cast over a glass substrate using a Teflon-coated Microm II Film Applicator (Paul N. Gardner Company, Inc., Pompano Beach, FL, USA) at a fixed height of 178 µm. The film was taken out of the glovebox and immediately submerged into the coagulation bath, where the solvent and the pore former diffused towards the non-solvent, leaving a composite polymer film. The coagulation bath was set at room temperature, measured to be 22 ± 2 °C.

#### 2.2.2. Fabrication of Thin-Film Composite Membranes by Interfacial Polymerization

A polyamide selective layer was formed on top of the nanocomposite supports. A 2.0 wt % MPD solution in DI water and a TMC solution (0.15 g TMC in 100 mL of n-hexane) were prepared and mixed for at least 3 h. Nanocomposite support coupons were taped on top of glass slides without drying, with the less porous side facing outward. The coupon was submerged into the MPD solution for 2 min, removed from the solution, and excess liquid was removed using a rubber roller. After that, the coupon was submerged into the TMC solution for 1 min, removed from the solution, and allowed to rest for 2 min. An annealing process described by Lind et al. [32] was used in which the membrane taped to the glass slide was submerged in water at 90 °C for 2 min. After annealing, the tape was removed and the TFC membrane was submerged for 2 min in 0.1 wt % sodium hypochlorite solution in water, and then into a 0.1 wt % sodium bisulfite solution in water for 30 s. Finally, the membrane was rinsed in water at 90 °C for 2 min and stored in DI water prior to testing.

### 2.3. Materials Characterization

#### 2.3.1. MWCNTs and HCNTs

Attenuated total reflectance Fourier-transform infrared spectroscopy (ATR-FTIR) was used to check for the presence of chemical groups after functionalization of CNTs. A Thermo Scientific bench-scale Nicolet 6700 FTIR was used, equipped with a Thermo Spectra Tech Endurance Foundation Series Diamond AT (Thermo Fisher Scientific, Waltham, MA, USA). For each measurement, 128 scans were performed at a resolution of 4 cm^−1^, always with auto-gain beam intensity.

SEM was used to measure the dimensions, and to visualize the morphology of the CNTs. Samples were dispersed, mounted, and sputtered with gold–palladium using an ANATECH HUMMER® 6.5 (Anatech Limited, Denver, NC, USA). A Hitachi S4800 field emission scanning electron microscope (Hitachi Limited, Tokyo, Japan) was used to create micrographs with an accelerating voltage of 20 kV.

#### 2.3.2. Nanocomposite Supports and TFC Membranes

Thickness was measured using a Mitutoyo 293-340-30 Digital Micrometer (Mitutoyo Corporation, Kawasaki, Japan) with a cylindrical borosilicate substrate between the probe and the membrane to distribute the load. Eight measurements were taken per support.

Sessile contact angle measurements were done using a KRÜSS DSA 10 Mk2 goniometer (KRÜSS GmbH, Hamburg, Germany) with Drop Shape Analysis software (version 1.80.0.2). Samples were attached to a microscope slide using double-sided tape, making sure to keep the surface flat. Prior to measurements, nanocomposite supports were pat-dried with a lint-free Kimwipe®, and they were left to dry further under ambient conditions for 4 h. Three-and-a-half microliters of DI water were placed onto the support and allowed to equilibrate for 30 s. Six measurements were made per sample. 

The porosity (*ε*) was estimated using mass differences between a wet membrane (*m_wet_*) and a dry membrane (*m_dry_*). DI water was used as the wetting agent because it does not swell the membrane, and it does not evaporate appreciably during the timeframe of the measurement. A wet sample was placed between lint-free Kimwipes® to absorb excess water on the surface, the sample was weighed, and then it was dried to a constant weight in an oven at 80 °C overnight. Equation (1) was used to estimate the porosity. Four measurements were conducted for each sample. The symbol *ρ* represents the density of each material.

(1) $$\varepsilon =\frac{\left(m_{wet}-m_{dry}\right)/\rho_{water}}{\frac{\left(m_{wet}-m_{dry}\right)}{\rho_{water}}+\frac{m_{dry}}{\rho_{Matrimid}}}$$

Tensile strength and Young’s modulus of the supports were measured using an INSTRON 1125 Universal Testing Machine (Instron, Norwood, MA, USA). A 2 kg load cell was used, the gap within the clamping device was kept at 100 mm, the width of samples was 10 mm, and the pulling rate was 10 mm min^−1^. These conditions were based on the ASTM D882-12 standard used for plastic sheeting with thickness below 1 mm [33]. Five measurements were made per sample. The balance load and distance between jaws were recorded.

The reduced modulus and the deformation of nanocomposite supports upon compression were evaluated using a TA Instruments TMA Q400 Machine (TA Instruments Inc., New Castle, DE, USA) with a penetration probe. The contact diameter of this probe was 0.89 mm, which applies a pressure of 16 bar when the force is 1 N. Two different two-stage compression programs were used. The first program consisted of a force ramp from 0.05 N to 1.2 N (maximum limit of the instrument) at 1 N min^−1^, a force release back to 0.05 N at the same rate, and a second compression to 1.2 N again at the same rate. The second program was similar to the first one, but 1 min of rest time was added between the force ramp changes. The objective of the second program was to evaluate the deformation behavior at constant compression stress. The temperature was initially set at 22 °C and held constant for 1.5 min before starting the force ramp. Four measurements were taken using wetted nanocomposite support samples, and the load and sample thickness (*h*) were recorded. The total test time was 3.5 min for the first program and 5.5 min for the second program. The reduced modulus was calculated as the initial slope when the plotting compressive stress (*σ*) versus the relative change in thickness of the support during the compression stage *i*, as shown in Equation (2).

(2) $$E_{r,i}=\frac{d\sigma_{i}}{d\left(∆h_{i}/h_{0,i}\right)}$$

ATR-FTIR with the previously mentioned instrument was used to observe changes in the nanocomposite support, due to the addition of treated CNTs. Four dry samples were analyzed per load and type of CNTs. Sixteen scans were performed at a resolution of 4 cm^−1^, always with auto-gain beam intensity.

SEM with the previously mentioned instrument was used to study the cross-sectional areas of different nanocomposite supports. Samples were flash-frozen in liquid nitrogen, cracked, mounted, and sputtered with gold-palladium. The accelerating voltage was 10 kV.

### 2.4. TFC Membrane Performance Testing

#### 2.4.1. Nanocomposite Support Pure Water Permeance

The pure water permeance of nanocomposite supports was assessed with direct-flow filtration using a Sterlitech HP4750 Stirred Cell (Sterlitech Corporation, Kent, WA). A support coupon was tested at four different pressures (up to 138 kPa). Measurements were done three times in the order of increasing pressure, then decreasing pressure, and finally increasing pressure again. The water flow rate was recorded over time, and the pure water permeance was calculated as the slope of the water flux versus the pressure plot. Three nanocomposite support coupons were tested per load and type of CNTs. The measurement duration was 35–40 min.

#### 2.4.2. Two-Stage Water Flux Measurements

Water flux changes during pressure step changes were measured in a home-built apparatus. The piping and instrumentation diagram was reported previously [34].

To determine the effect of the addition of CNTs on water flux upon compression, a 2000 ppm solution of NaCl was recirculated through the membrane cell with an installed membrane coupon at 1 L min^−1^ and 862 kPa. Permeate flow rate was measured until it stabilized (i.e., a constant mass flow rate was observed for 15 min), and then the flow rate was measured over the course of two pressure cycles. Each cycle comprised the operation of the cell at *P*_1_ = 1380 kPa for 15 min, reducing the pressure to *P*_2_ = 862 kPa for 15 min, and returning the pressure to 1380 kPa to start the next cycle. These pressure values were selected because they are above the osmotic pressure of the feed solution (~170 kPa), and compared with the stress values applied in the TMA penetration test, which was used at its upper limit. Five liters of fresh solution were used to avoid concentration build-up and fouling. Permeate flow rate was divided by the exposed membrane area (~610 mm^2^) to obtain the water flux (*J_w_*). In these experiments, the starting time (*t =* 0 min) was defined as 15 minutes before the first pressure cycle. At this time, permeate was collected for salt rejection measurements. Three TFC membrane coupons per load of CNTs were tested. A similar experiment at one pressure has been used by Pendergast et al. [35] to measure the loss of permeability due to physical compaction in composite supports comprising PSf and zeolite A.

The experiment described above was designed to recreate a similar compressive load to the one used in the TMA penetration tests. The purpose of the first pressure step was to condition the membrane by compressing the support layer and fully wetting the structure, whereas the purpose of the second pressure step was to measure the membrane performance. The change in the water permeance (*A*, Equation (3)) for each measurement was used to determine the changes due to compaction. The compared values were the average permeance in the second cycle at *P*_1_ and *P*_2_. The permeance was selected instead of water flux to compare results at different pressures. Equations (4) and (5) were used to compute the NaCl rejection (*R*) and the change in permeance after compression. In these equations, *i* is the van ′t Hoff factor, *c* is the concentration of sodium chloride of the feed and permeate (measured by conductivity), *R_u_* is the universal gas constant, *T* is the test temperature, and *P* is the pressure at the level *j* (1, 2) in the cycle *k* (first or second). The measurement duration was 90–100 min.

(3) $$A_{P_{j,k}}=J_{w,\;P_{j,k}}/\left(P_{j,k}-iR_{u}T\left(c_{f}-c_{p}\right)\right)$$

(4) $$R=1-\frac{c_{p}}{c_{f}}$$

(5) $$\mathrm{Relative}\;\mathrm{permeance}\;\mathrm{decrease}=\left(A_{P_{2,2}}/A_{P_{1,2}}-1\right)\times 100\%$$

## 3. Results and Discussion

### 3.1. CNTs Synthesis

**Figure 2:**
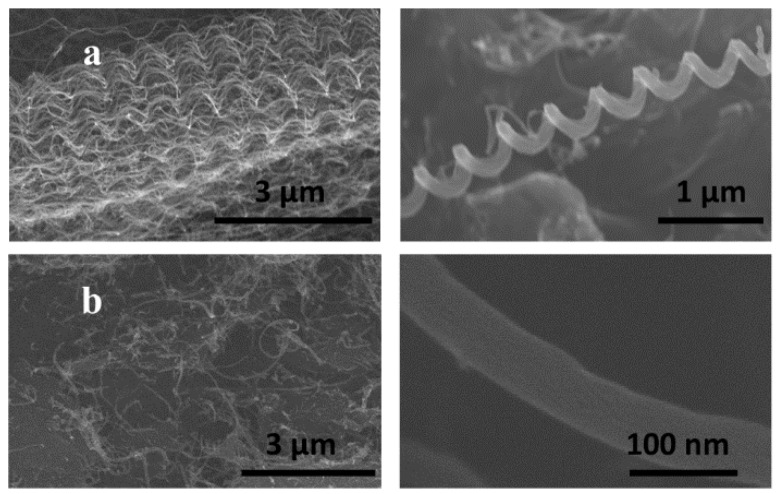
Scanning-electron microscopy (SEM) images of (**a**) hellicaly-coiled carbon nanotubes (HCNTs) and (**b**) multi-walled carbon nanotubes (MWCNTs).

Figure 2a,b show SEM images of HCNTs and MWCNTs. MWCNTs have a cylindrical shape, with random slight curves; whereas HCNTs show a coiled tube structure. By analyzing these images, it was determined that the MWCNTs are short (less than 20 μm) compared to HCNTs (approximately 100 µm). MWCNTs have a diameter of ~80 nm, while HCNTs are much narrower at only 20 nm with a pitch ranging from 400 to 600 nm. The diameter of the HCNT coils is commensurate with the pitch, being ~400 nm wide. Despite differences in their shape, both show a high aspect ratio that is beneficial for the creation of structural composites. Figure S1 shows IR transmission spectra of CNTs before and after functionalization.

### 3.2. Nanocomposite Support and TFC Membrane Characteristics

**Figure 3:**
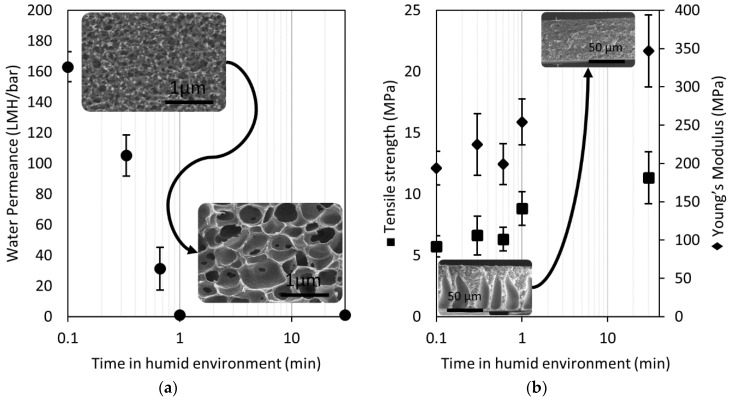
(**a**) Permeance of Matrimid films cast at different times in a humid environment. (**b**) Tensile strength of Matrimid films cast at different times in a humid environment. SEM image insets show the cross-sections of the Matrimid films.

Figure 3a presents the water permeance (in L·m^−2^·h^−1^·bar^−1^, LMH·bar^−1^) of Matrimid films cast at 32% relative humidity (RH) and different times before wet phase inversion. The permeance decreases considerably by contacting the polymer solution film with a humid environment before immersion. This result could be explained by the reduced number of interconnections among cells inside the film cross-section and the clogging of pores at the top surface. Nonetheless, Lee et al. [14] have shown that supports fabricated via wet phase inversion with larger surface pore size (higher water permeance) have a lower strength and Young’s modulus.

Figure 3b shows the tensile strength and Young’s modulus of Matrimid films cast at 32% RH and at different times before wet phase inversion. Both mechanical properties increase by contacting the polymer solution film with a humid environment before immersion. In this case, the elimination of macrovoids is considered to be responsible for the improvement in the mechanical strength of the membrane. Similarly, Guillen et al. [36] showed that films made with a PSf solution in DMF can readily absorb atmospheric water (nonsolvent), which causes the formation of a barrier that prevents macrovoid formation. These observations support the idea that a reduction in the concentration gradient of the solvent between the interface of the polymer solution and nonsolvent promotes the formation of macrovoid-free films. A TFC membrane support layer can be tuned to achieve a strong, macrovoid-free film; however, it must also be a fully interconnected structure to have an acceptable water permeance. In this work, we decided to cast films using low RH by operating in a nitrogen-purged glovebox to produce supports with interconnected pores. Although it was not within the scope of this project, we believe that the RH and the amount of nonsolvent can be tuned to obtain porous polymer films with the above-mentioned characteristics.

**Figure 4:**
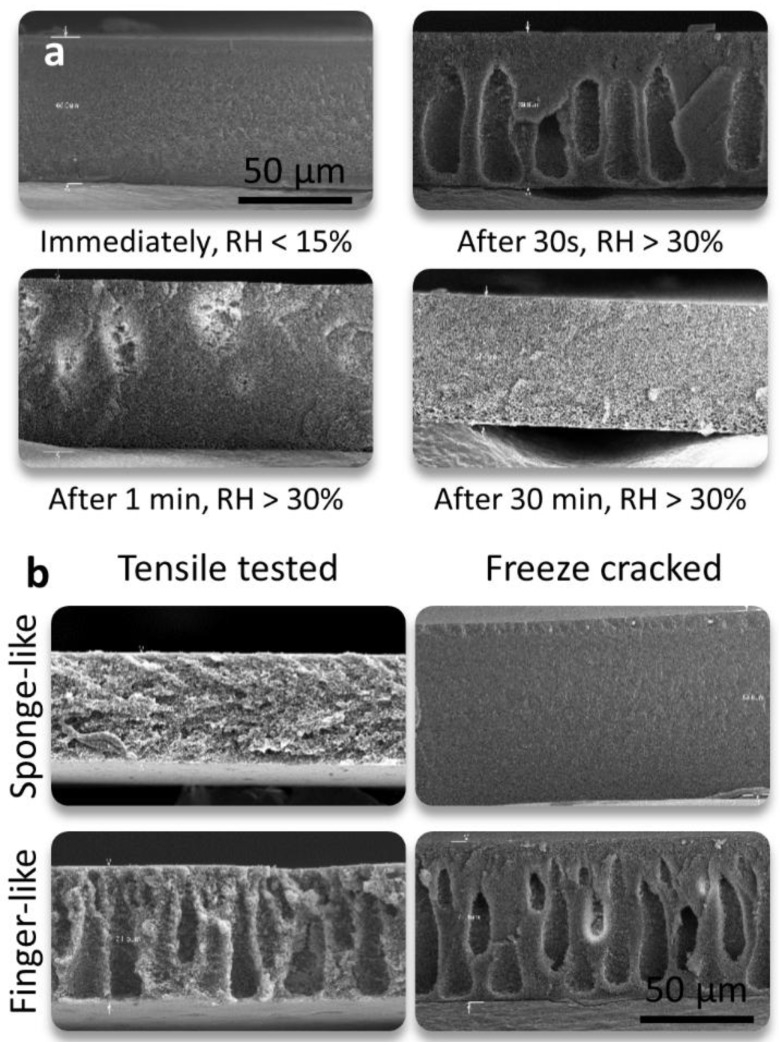
(**a**) Cross-sectional SEM images of Matrimid films cast at different times in a humid environment. (**b**) SEM images of films with sponge-like and finger-like structures after tensile testing and freeze-cracking.

Figure 4a shows SEM images of the nanocomposite support cross sections. The structure depends strongly on the relative humidity and times before the wet phase inversion. At low relative humidity and fast immersion, the cross-section tends to be uniform, and it shows a sponge-like structure. When the RH is above 30% and the time between casting and phase inversion is 30–60 s, the formation of macrovoids is observed. However, if the film is left in a humid environment for 30 min prior to phase inversion, another fully sponge-like structure is observed. For the latter case, the pores in the cross section appear to be larger and less interconnected than in the first case. Because the polymer concentration and casting thickness was kept constant, it was expected and observed that macrovoids would lead to an increased thickness. Figure 4b shows the SEM images of the nanocomposite support cross-sections in the zone of failure after tensile testing and after freeze cracking. For both supports, there is a decrease in the thickness from before tensile testing to after testing, due to the tensile stress. Additionally, there is a generalized increase in the roughness in the sponge-like cross-section due to failure, whereas this effect is localized around the macrovoids in the finger-like structure. For supports with finger-like structures, there is a reduction in the effective cross-sectional area where the load is applied during a tensile test. This implies that the stress on the sample is greater at the same load for supports with a finger-like structure, making them more susceptible to failure.

Figure S2 (Supplementary Materials) presents spectra obtained by ATR-FTIR of nanocomposite supports with different CNTs loads. It has been argued that favorable interactions of CNT surface functional groups with water increases the flux through the films [16,18,20]; however, in our case, the low amount of CNTs (≤2 wt %) added to the films showed no noticeable changes in the IR spectra (i.e. chemistry) of the nanocomposite supports.

**Figure 5:**
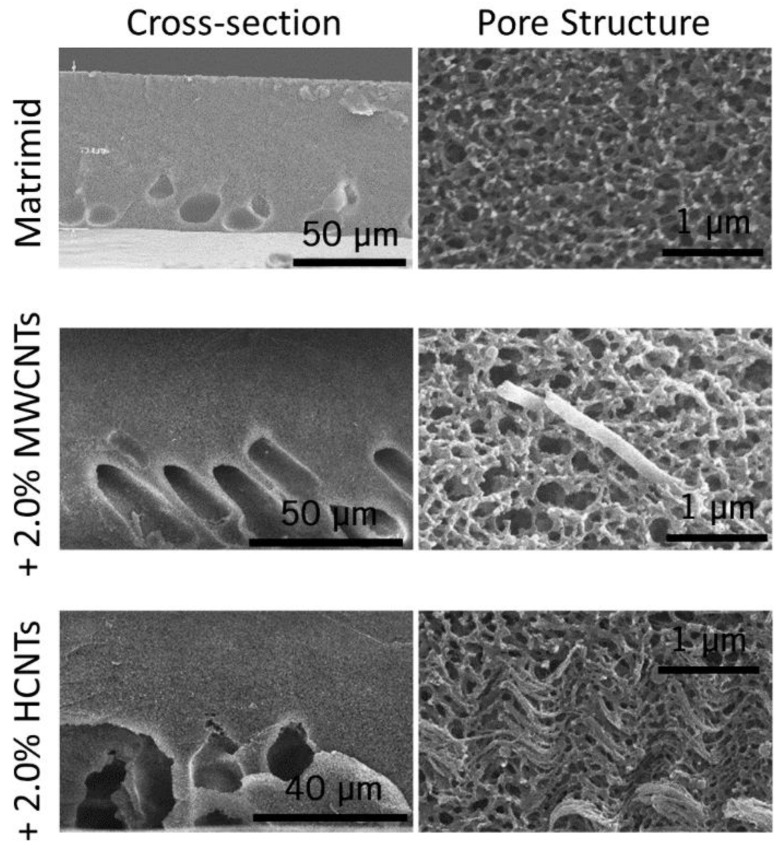
Cross-sectional SEM images of supports as fabricated.

Figure 5 shows SEM images of the cross-section and pore structure of the fabricated Matrimid films (no CNT load) and nanocomposite supports. Matrimid films typically had fewer (or no) macrovoids compared to nanocomposite supports. It has been proposed that particle addition in a nanocomposite support promotes the formation of macrovoids, due to hindered diffusion of the solvent, created by the fillers, during the phase separation [37]. Accordingly, we believe that the formation of macrovoids in our nanocomposite supports can be attributed to the CNT fillers. On the other hand, the pore structure was fully interconnected and similar for all cases. This was expected based on findings from Figure 3a, as the casting was done at low humidity. CNTs were seen at different positions along the cross-section, and they were usually parallel to the plane of the film. This orientation results from the drag of the doctor blade during the polymer solution casting. Some agglomeration was seen in films with HCNT loading, probably because of the higher contact area and entanglement between individual CNTs, due to the coiled nature of their growth.

Table 1 presents the average values of thickness, porosity, contact angle, and water permeance of the nanocomposite supports. Thickness and porosity increased upon addition of CNTs. We used a constant amount of polymer to create a constant area of support; therefore, these changes are attributed to the formation of macrovoids. The measured contact angle of the supports showed no significant changes or trend upon addition of CNTs, mostly having an average value of 80°, similar to the Matrimid films with no CNTs. This was expected, as no changes in chemistry were observed with IR in the nanocomposite support, and any variability obtained could be attributed to differences in the surface roughness and the surface porosity [38]. The pure water permeance (PWP) of the nanocomposite supports was on average above 200 LMH·bar^−1^. Also, the PWP of the nanocomposite supports were not different at a confidence interval of 95% when compared to the control (Matrimid). However, an increasing trend in PWP was observed with increasing MWCNT loading, consistent to the observations of Kim et al., who added MWCNTs to the PSf support [18]. Nevertheless, PWP values for the supports were two orders of magnitude higher than TFC membranes made with MPD and TMC polyamide chemistry [32]; thus, any variation in the water flux through TFC membranes cast using these nanocomposite supports would be due to variations of the skin layer and/or differences in deformation of the support due to compression.

A model was developed to describe the Young’s modulus of the porous polymer supports. Parameters for the model were determined by fitting experimental data for Young’s modulus and porosity. The Supplementary Materials document contains the model derivation. This model provides a range of values for the Young’s modulus. Initially, values for the upper and lower bounds were calculated using the Rule of Mixtures for the cases of axial and transverse loading [31]. Figure S3 shows a schematic representation used for the derivation of the model. Then, these bounds were corrected, taking into account the aspect ratio of the filler and the porosity of the film. Equations (6) and (7) are the derived upper and lower bounds for the Young’s modulus. Here, *f* is the volumetric fraction of filler, *E* is the Young’s modulus of the matrix and the filler, *η* is the contact efficiency associated with the aspect ratio of the filler, *φ* is the porosity of the membrane, and *n* is an adjustable parameter. Table S1 contains the nomenclature used for the model derivation. The predicted value represents the harmonic average between the upper and lower bounds.

(6) $$\;E_{\mathrm{membrane}}^{\mathrm{upper}-\mathrm{bound}}=\left(\left(1-f\right)E_{\mathrm{matrix}}+\eta_{Aspect}fE_{\mathrm{filler}}\;\right){\left(1-\phi \right)}^{n}\;$$

(7) $$\;E_{\mathrm{membrane}}^{\mathrm{lower}-\mathrm{bound}}={\left(\frac{1-f}{E_{\mathrm{matrix}}}+\frac{f}{\eta_{Aspect}E_{\mathrm{filler}}}\right)}^{-1}{\left(1-\phi \right)}^{n}\;$$

**Figure 6:**
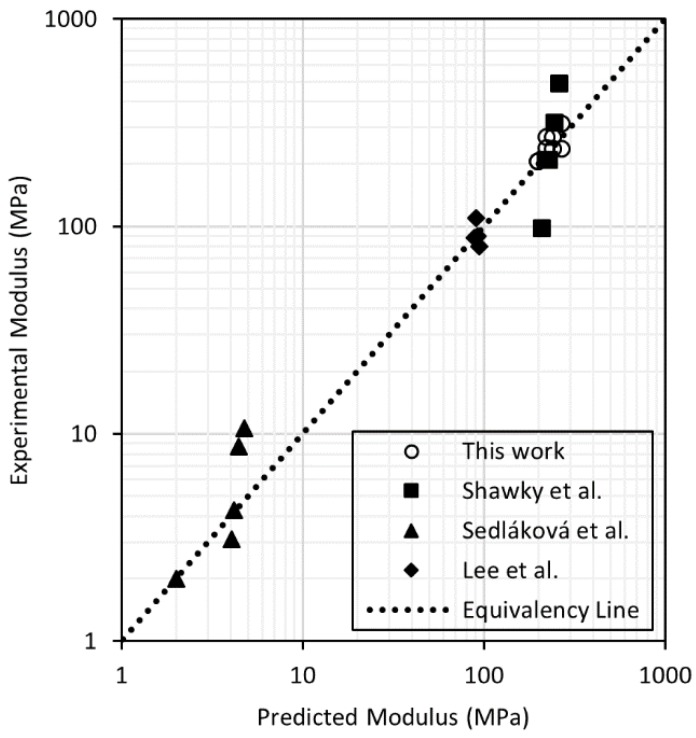
Reported [14,21,39] and predicted Young’s modulus of nanocomposite supports fabricated using polymer and carbon nanomaterials.

Firstly, the model was used to fit the results obtained by Sedláková et al. [39] for CNT/ethylene–octene copolymer membranes used for gas and vapor separations. Figure 6 shows that the values of Young’s modulus of the membranes made by Sedláková et al. had a slight positive deviation from the predicted value, indicating a good contact and orientation, probably due to the non-porous nature of the films fabricated. Values for the nanocomposite supports fabricated in this work and membranes prepared by Shawky et al. [21] using CNTs and aromatic polyamide were distributed around the predicted harmonic average Young’s modulus. A similar observation was made using Young’s modulus results as reported by Lee et al. [14] using PSf and thickness-controlled graphene oxide. In the latter two cases, a porosity of 70% was used, and a value for *n* was regressed from fits to experimental data. The values for *n* were 2.33 and 2.74 for the data obtained by Shawky et al. and Lee et al., whereas our nanocomposite values led to a value of 2.23. Differences in these values were most likely due to the different methods used for nanocomposite support fabrication. We used wet phase inversion, while Shawky et al. used solvent evaporation. Additionally, the higher variation in the results reported by Shawky et al. can be attributed to the use of a radical initiator during the mixing of the CNTs and the polymer to create covalent bonds with the filler, something that the proposed model does not consider. On the other hand, Lee et al. used wet phase inversion with PSf and NMP, which produced nanocomposite supports with macrovoids. These macrovoids have a detrimental effect on the modulus (i.e., higher *n* value).

Overall, this model showed good agreement with experimental values of the Young’s modulus of porous films, and it can be a useful tool to predict mechanical properties of polymeric membranes. Further validation of the model will require additional mechanical property data (tensile strength and Young’s modulus) for porous membranes and membrane composites, which are ultimately important to understand membrane performance and failure mechanisms [22]. The collection of such data using a method such as ASTM D882-12 should become standard practice for new membrane development efforts. The porosity, orientation, and the amount of filler used to make composite membranes also should be reported in new membrane development efforts. Additional experimental measurements would reveal common values for *n* for different polymer-filler combinations, improving the predictive nature of the model.

**Figure 7:**
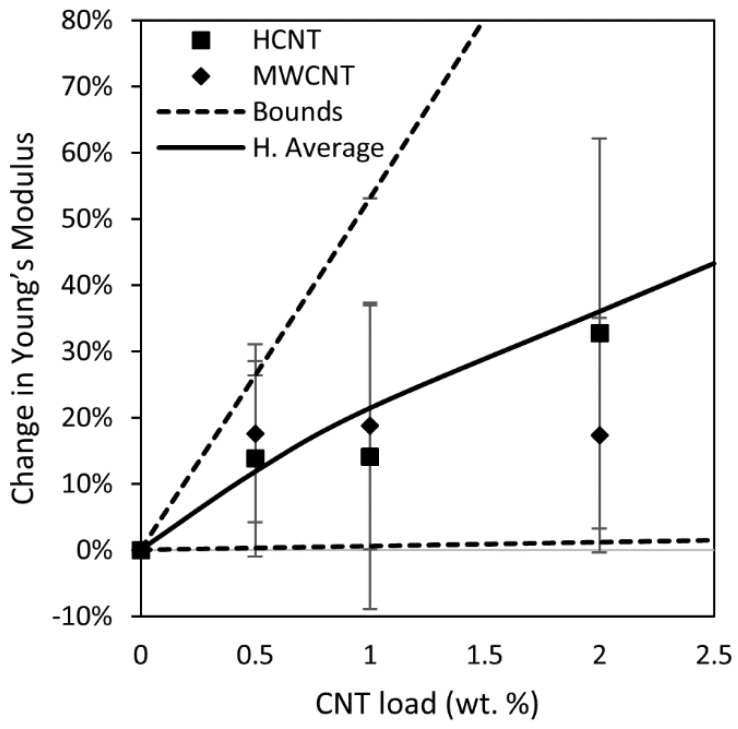
Relative change of the Young’s Modulus upon addition of CNTs to form the nanocomposite support. Dashed curves represent the upper and lower bounds calculated using Equations (6) and (7). The solid curve represents the harmonic average.

Figure 7 presents the improvement in the Young’s Modulus of nanocomposite supports compared to the Matrimid films, as well as the predicted upper and lower bounds. Both the variance and modulus increased upon addition of CNTs. The variance increased because the number of possible orientations of the CNTs increases with increasing load. The experimental results best matched the harmonic average of the bounds, in comparison to the arithmetic and geometric averages. Finally, no significant difference between the types of CNTs was found, most likely because both materials have similar modulus values, their aspect ratios are sufficiently high to have an efficient stress transfer, and the orientation of both CNTs types appeared to be parallel to the plane of the tensile test [40].

**Figure 8:**
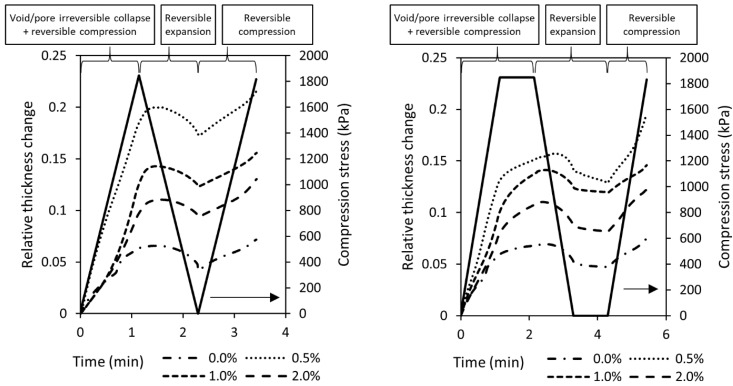
Example of typical TMA penetration experiment compressive stress and relative thickness change results. (**Left**) without rest times and (**right**) with a rest time of 1 min in between force ramps. Experiments start at stress and the relative thickness change equals zero.

Figure 8 illustrates typical data from TMA penetration experiments that show relative changes in thickness and compression stress over the test time period. On the right are experiments with different CNT loads and a 1 min rest time before force ramps. On the left are results without a rest time. The reduced modulus was evaluated using Equation (2) for each penetration step. Reduced modulus values during the second penetration step were always higher than the first penetration step, demonstrating that membranes undergo irreversible deformation during operation, most likely related to the collapse of macrovoids and some irreversible pore collapses. Thickness changes due to compression stresses are also visible in Figure S4 (Supplementary Materials), which show cross-sectional SEM images before and after two-stage pressure stepping water flux measurements. During the first penetration step, the thickness change was on average 12% (~10 µm). CNT-free samples showed the lowest change in thickness during this step, due to their lower content of macrovoids. Experiments with rest time showed that slow deformation can continue when the compression stress is kept constant. Partial elastic deformation of the nanocomposite supports was observed as a thickness increase of ~2 µm in all samples after releasing the compressive stress. These experiments suggested that ~20% of the initial deformation is reversible. Therefore, it is expected that during the first penetration, the reduced modulus would be related largely to the overall cross-sectional structure; whereas during the second compression, the reduced modulus would be related largely to the material composition and porosity. Additionally, these thickness changes corresponded to porosity reductions of 3-6% after the first compression. This range was comparable to the uncertainty (95% confidence level) of the porosity measurement (Table 1) which did not show a correlation with the nanocomposite support PWP. Therefore, we believe that decrease in the water permeance of TFC membranes after the initial compression was not likely to be associated with the macrovoids collapsing.

**Figure 9:**
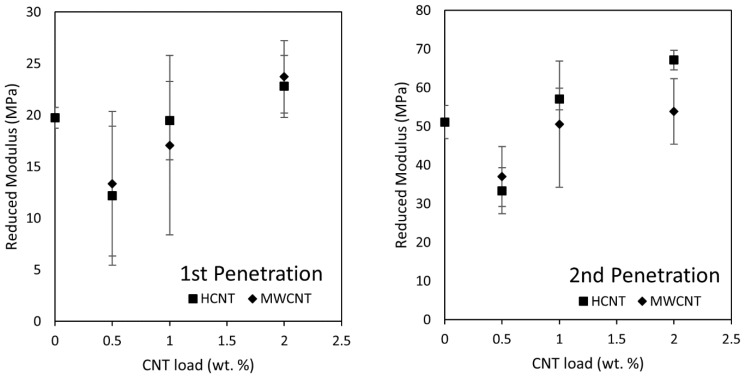
Reduced modulus during the first (**left**) and second (**right**) penetration of Matrimid/CNT nanocomposite supports fabricated using HCNTs and MWCNTs.

Figure 9 shows the reduced modulus of nanocomposite supports fabricated at different loads of MWCNTs and HCNTs. On the left are the values during the first penetration, which show no significant difference between the reduced moduli obtained with different types of CNTs. This finding is consistent with the assertion that the overall cross-section structure largely controls the initial mechanical behavior of the supports. On the right are the values during the second penetration. A lower reduced modulus was observed for samples with 0.5 wt % CNT compared with CNT-free supports (control). This is attributed to differences in the support structure. CNT-free supports are almost fully sponge-like; whereas, CNT-loaded supports have particle-induced macrovoids. At 0.5 wt % CNT, there is insufficient CNT loading to overcome the detrimental effects of macrovoid collapse, which does not occur for the CNT-free supports. However, there is an increasing trend in the reduced modulus from 0.5 to 2.0 wt % of CNTs, equivalent to an increase of 75% within this range, and a net increment of 18% relative to the CNT-free control. These findings support the idea that the addition of CNTs has the capability of increasing structural stiffness of the nanocomposite support; however, the fabrication of fully sponge-like microstructures (such as the films without CNTs) also has a pronounced effect on the mechanical properties. Just like the tensile test results, the difference in the support stiffness between using MWCNTs and HCNTs was not significant, compared to the load of CNTs. Therefore, the following two-stage water flux measurement were done solely with MWCNTs, because they are available commercially.

### 3.3. TFC Membrane Fabrication and Performance

Interfacial polymerization was used to form a polyamide skin layer on the top of nanocomposite supports. Figure S5 (left) in Supporting Information shows an ATR-FTIR spectrum of a nanocomposite support (bottom) and the active layer (top). Three peaks appeared after the polymerization. These were at 1659 cm^−1^ and 1543 cm^−1^, assigned to amide bond stretching, and a peak at 1611 cm^−1^ was assigned to aromatic ring stretching [41]. Figure S5 (right) in Supporting Information shows SEM images of the top surface of TFC membranes and the nanocomposite support. The supports have pores of less than 50 nm diameter, whereas the TFC membranes had a characteristic ridge and valley structure of the polyamide formed from interfacial polymerization of MPD and TMC. Figure S6 shows the SEM images of the top surface after interfacial polymerization. No significant differences in the morphology of the polyamide layer were found using different loads of CNTs in the nanocomposite support.

**Figure 10:**
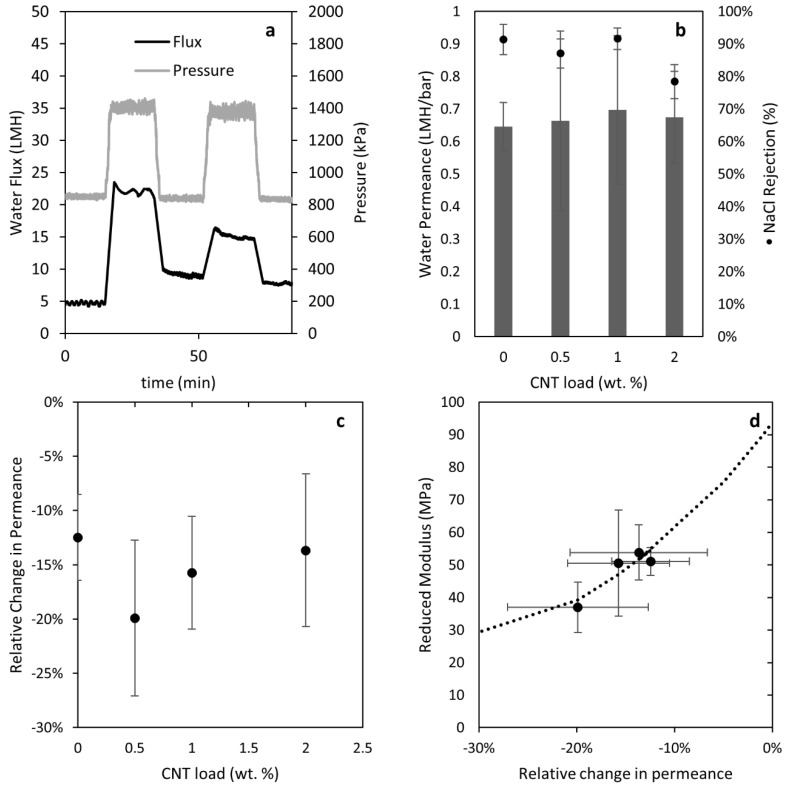
(**a**) Experimental results of two-stage pressure stepping water flux measurements for a TFC membrane with a 2.0% CNT-loaded nanocomposite support. (**b**) Water permeance and NaCl rejection of TFC membranes using nanocomposite supports with different CNT loads. (**c**) Relative decrease in permeance of TFC membranes using nanocomposite supports with different CNT loads. (**d**) Relation between the reduced modulus measurements and the relative decrease in permeance (line added to guide the reader).

Figure 10a shows representative data from a two-stage pressure stepping water flux measurement. It is important to note that, in some measurements, the flux at the beginning of the experiment was lower than the one at the end of the experiment, with both at the same pressure. We attribute this to pressure-induced wetting, and we do not expect or observe that lowering the pressure would result in restoring to the original state [42]. The water permeance at each pressure and stage was calculated as an average over the 15 min length of each pressure step. Figure 10b shows the values of water permeance *A* and NaCl rejection *R* estimated at the end of the experiment. Similar water permeance was observed using different nanocomposite supports, and all values were much lower than the nanocomposite support PWP, because the skin layer produces the main water flow resistance in a TFC membrane. We used 75% NaCl rejection (nanofiltration) as an acceptance criterion for the formation of intact polyamide thin films. Yip et al. [43] studied a similar post-treatment to the one used here, and found an increase in the variability of NaCl permeability after treatment. Therefore, we attribute variations in the NaCl rejection to the hand-casting procedure, rather than the change in stiffness of the nanocomposite supports. Statistical analysis failed to reject the hypothesis that the NaCl rejection was equal when comparing the 0% and 2% CNT loading at 95%. Tables S2 and S3 shows statistical analysis results comparing the water permeance and salt rejection of the nanocomposite supports to the Matrimid support. Figure 10c shows results for the relative change in permeance. Loss of permeance was reduced by increasing the load of CNT from 0.5 to 2.0%. This behavior is consistent with the results observed for the reduced modulus measured by the TMA experiments (Figure 9). Figure 10d correlates the relative change in permeance to the reduced modulus for CNT-loaded supports. The correlation coefficient was calculated to be 91.8%. By increasing the reduced modulus, we can limit decreases in water permeance due to support compression. Lonsdale et al. [44] and Pendergast et al. [30] have previously proposed that changes in the pore size of supports due to compaction could explain the changes in water flux. Here, we show that such changes can be observed using two-stage TMA penetration tests, and that the reduced modulus serves as an indicator of the support stiffness. Additionally, we demonstrate that increasing the mechanical stiffness of the support is an effective strategy for preserving water permeance at high pressure in TFC membranes.

## 4. Conclusions

Nanocomposite supports for TFC membranes made from Matrimid and helically coiled or straight multiwall carbon nanotubes were fabricated via wet phase inversion. Young’s modulus increased by 20% on average upon the addition of 2.0% CNTs, as did the measurement variance. A model is proposed that predicts the increased modulus and implies that the increased variance is a result of the random orientation of the CNTs within the nanocomposite support. With further validation, this model can be used for membrane design by estimating the required additive load in the nanocomposite support and the maximum acceptable porosity for attaining the targeted mechanical stiffness. While a 0.5% loading of CNTs caused macrovoids that decreased the structural integrity by 35%, adding 2.0% loads of CNTs compensated for this effect, and resulted in a net increase of 18% in structural stiffness. Additionally, it was found that an increased compressive stiffness of the CNT-loaded nanocomposite supports reduced the water permeance losses associated with the compression of the support. These findings support the idea that increasing the mechanical stiffness of the TFC membrane nanocomposite supports is an effective strategy for enhancing water production in desalination operations.

## Figures and Tables

**Table 1:** Properties of nanocomposite supports. The uncertainty values represent a 95% confidence interval.

Support	Thickness (µm)	Porosity (%)	Contact Angle (°)	Pure Water Permeance (LMH·bar^–1^)
Matrimid	72 ± 5	56 ± 6	81 ± 1	208 ± 33
MWCNTs 0.5	115 ± 2	76 ± 2	78 ± 6	217 ± 41
MWCNTs 1.0	74 ± 8	69 ± 4	84 ± 2	220 ± 23
MWCNTs 2.0	84 ± 4	72 ± 4	86 ± 4	266 ± 25
HCNTs 0.5	93 ± 4	74 ± 1	78 ± 6	228 ± 97
HCNTs 1.0	94 ± 12	74 ± 1	78 ± 4	379 ± 199
HCNTs 2.0	99 ± 15	74 ± 2	77 ± 4	136 ± 48

## Supplementary Materials

The following are available online at http://www.mdpi.com/2077-0375/8/4/111/s1, Figure S1: Typical IR spectra of pristine and functionalized CNTs, Figure S2: ATR-FTIR spectra of composite supports with different CNT loads, Figure S3: (Left) Schematic representation of a fully solid composite material, where the matrix is a polymer and the filler is a carbonaceous material. (Right) Control volume describing the interface between the matrix and the filler, where the total height is 1 unit, and the component heights are proportional to their volume fractions, Figure S4: Typical cross-sectional SEM images of TFC membranes using composite supports before and after two-stage pressure stepping water flux measurements, Figure S5: (Left) ATR-FTIR spectra comparison of TFC membranes and composite support (polyamide formation). (Right) SEM imaging of the top surface of the TFC membrane, Figure S6: SEM images of the top surface of the TFC membranes after interfacial polymerization on nanocomposite supports with different CNT loading, Table S1: Nomenclature used in model, Table S2: Statistical analysis of the effect of CNT loading of nanocomposite supports in the water permeance (A) of the respective TFC membranes, Table S3: Statistical analysis of the effect of CNT loading of nanocomposite supports in the NaCl rejection of the respective TFC membranes.

## References

[1] Chung T.S., Luo L., Wan C.F., Cui Y., Amy G. (2015). What is next for forward osmosis (FO) and pressure retarded osmosis (PRO). Sep. Purif. Technol..

[2] Elimelech M., Phillip W. (2011). The future of seawater desalination: Energy, technology, and the environment. Science.

[3] Achilli A., Childress A.E. (2010). Pressure retarded osmosis: From the vision of Sidney Loeb to the first prototype installation—Review. Desalination.

[4] Kim J., Park M., Shon H.K., Kim J.H. (2016). Performance analysis of reverse osmosis, membrane distillation, and pressure-retarded osmosis hybrid processes. Desalination.

[5] Logan B.E., Elimelech M. (2012). Membrane-based processes for sustainable power generation using water. Nature.

[6] Paul M., Jons S.D. (2016). Chemistry and fabrication of polymeric nanofiltration membranes: A review. Polymer.

[7] Geise G.M., Paul D.R., Freeman B.D. (2014). Fundamental water and salt transport properties of polymeric materials. Prog. Polym. Sci..

[8] Tiraferri A., Yip N.Y., Straub A.P., Castrillon S.Ro., Elimelech M. (2013). A method for the simultaneous determination of transport and structural parameters of forward osmosis membranes. J. Memb. Sci..

[9] Xu G.R., Wang J.N., Li C.J. (2013). Strategies for improving the performance of the polyamide thin film composite (PA-TFC) reverse osmosis (RO) membranes: Surface modifications and nanoparticles incorporations. Desalination.

[10] Ismail A.F., Padaki M., Hilal N., Matsuura T., Lau W.J. (2015). Thin film composite membrane—Recent development and future potential. Desalination.

[11] Manawi Y., Kochkodan V., Hussein M.A., Khaleel M.A., Khraisheh M., Hilal N. (2016). Can carbon-based nanomaterials revolutionize membrane fabrication for water treatment and desalination?. Desalination.

[12] Alberto M., Bhavsar R., Luque-Alled J.M., Prestat E., Gao L., Budd P.M., Vijayaraghavan A., Szekely G., Holmes S.M., Gorgojo P. (2018). Study on the formation of thin film nanocomposite (TFN) membranes of polymers of intrinsic microporosity and graphene-like fillers: Effect of lateral flake size and chemical functionalization. J. Memb. Sci..

[13] Lai G.S., Lau W.J., Goh P.S., Ismail A.F., Tan Y.H., Chong C.Y., Krause-Rehberg R., Awad S. (2018). Tailor-made thin film nanocomposite membrane incorporated with graphene oxide using novel interfacial polymerization technique for enhanced water separation. Chem. Eng. J..

[14] Lee J., Jang J.H., Chae H.-R., Lee S.H., Lee C.-H., Park P.-K., Won Y.-J., Kim I.-C. (2015). A facile route to enhance the water flux of a thin-film composite reverse osmosis membrane: Incorporating thickness-controlled graphene oxide into a highly porous support layer. J. Mater. Chem. A.

[15] Zhao H., Qiu S., Wu L., Zhang L., Chen H., Gao C. (2014). Improving the performance of polyamide reverse osmosis membrane by incorporation of modified multi-walled carbon nanotubes. J. Memb. Sci..

[16] Son M., Choi H.g., Liu L., Celik E., Park H., Choi H. (2015). Efficacy of carbon nanotube positioning in the polyethersulfone support layer on the performance of thin-film composite membrane for desalination. Chem. Eng. J..

[17] Son M., Park H., Liu L., Choi H., Kim J.H., Choi H. (2016). Thin-film nanocomposite membrane with CNT positioning in support layer for energy harvesting from saline water. Chem. Eng. J..

[18] Kim E.S., Hwang G., El-Din M.G., Liu Y. (2012). Development of nanosilver and multi-walled carbon nanotubes thin-film nanocomposite membrane for enhanced water treatment. J. Memb. Sci..

[19] Song X., Wang L., Tang C.Y., Wang Z., Gao C. (2015). Fabrication of carbon nanotubes incorporated double-skinned thin film nanocomposite membranes for enhanced separation performance and antifouling capability in forward osmosis process. Desalination.

[20] Lee J., Ye Y., Ward A.J., Zhou C., Chen V., Minett A.I., Lee S., Liu Z., Chae S.R., Shi J. (2016). High flux and high selectivity carbon nanotube composite membranes for natural organic matter removal. Sep. Purif. Technol..

[21] Shawky H., Chae S.R., Lin S., Wiesner M.R. (2011). Synthesis and characterization of a carbon nanotube/polymer nanocomposite membrane for water treatment. Desalination.

[22] Wang K., Abdalla A.A., Khaleel M.A., Hilal N., Khraisheh M.K. (2017). Mechanical properties of water desalination and wastewater treatment membranes. Desalination.

[23] Arash B., Wang Q., Varadan V.K. (2014). Mechanical properties of carbon nanotube/polymer composites. Sci. Rep..

[24] Coleman J.N., Khan U., Blau W.J., Gun’ko Y.K. (2006). Small but strong: A review of the mechanical properties of carbon nanotube—Polymer composites. Carbon.

[25] Shokrieh M.M., Rafiee R. (2010). A review of the mechanical properties of isolated carbon nanotubes and carbon nanotube composites. Mech. Compos. Mater..

[26] Yu M.-F. (2004). Fundamental mechanical properties of carbon nanotubes: Current understanding and the related experimental studies. J. Eng. Mater. Technol..

[27] Cao A., Dickrell P.L., Sawyer W.G., Ghasemi-Nejhad M.N., Ajayan P.M. (2005). Super-compressible foamlike carbon nanotube films. Science.

[28] Daraio C., Nesterenko V.F., Jin S., Wang W., Rao A.M. (2006). Impact response by a foamlike forest of coiled carbon nanotubes. J. Appl. Phys..

[29] Han G., Zhang S., Li X., Chung T.S. (2013). High performance thin film composite pressure retarded osmosis (PRO) membranes for renewable salinity-gradient energy generation. J. Memb. Sci..

[30] Pendergast M.T.M., Nygaard J.M., Ghosh A.K., Hoek E.M.V. (2010). Using nanocomposite materials technology to understand and control reverse osmosis membrane compaction. Desalination.

[31] Askeland D.R., Wright W.J. (2013). Essentials of Materials Science and Engineering.

[32] Lind M.L., Suk D.E., Nguyen T.V., Hoek E.M.V. (2010). Tailoring the structure of thin film nanocomposite membranes to achieve seawater RO membrane performance. Environ. Sci. Technol..

[33] ASTM International (2012). ASTM D882: Standard Test Method for Tensile Properties of Thin Plastic Sheeting.

[34] Idarraga-Mora J.A., Ladner D.A., Husson S.M. (2018). Thin-film composite membranes on polyester woven mesh with variable opening size for pressure-retarded osmosis. J. Memb. Sci..

[35] Pendergast M.T.M., Ghosh A.K., Hoek E.M.V. (2013). Separation performance and interfacial properties of nanocomposite reverse osmosis membranes. Desalination.

[36] Guillen G.R., Ramon G.Z., Kavehpour H.P., Kaner R.B., Hoek E.M.V. (2013). Direct microscopic observation of membrane formation by nonsolvent induced phase separation. J. Memb. Sci..

[37] Husain S., Koros W.J. (2009). Macrovoids in Hybrid Organic/Inorganic Hollow Fiber Membranes. Ind. Eng. Chem. Res..

[38] Wang Y., Ou R., Ge Q., Wang H., Xu T. (2013). Preparation of polyethersulfone/carbon nanotube substrate for high-performance forward osmosis membrane. Desalination.

[39] Sedláková Z., Clarizia G., Bernardo P., Jansen J.C., Slobodian P., Svoboda P., Kárászová M., Friess K., Izak P. (2014). Carbon nanotube- and carbon fiber-reinforcement of ethylene-octene copolymer membranes for gas and vapor separation. Membranes.

[40] Volodin A., Ahlskog M., Seynaeve E., van Haesendonck C., Fonseca A., Nagy J.B. (2000). Imaging the elastic properties of coiled carbon nanotubes with atomic force microscopy. Phys. Rev. Lett..

[41] Tang C.Y., Kwon Y.N., Leckie J.O. (2009). Effect of membrane chemistry and coating layer on physiochemical properties of thin film composite polyamide RO and NF membranes. I. FTIR and XPS characterization of polyamide and coating layer chemistry. Desalination.

[42] Smirnov S., Vlassiouk I., Takmakov P., Rios F. (2010). Water Confinement in Hydrophobic Nanopores. Pressure-Induced Wetting and Drying. ACS Nano.

[43] Yip N.Y., Tiraferri A., Phillip W.A., Schiffman J.D., Hoover L.A., Kim Y.C., Elimelech M. (2011). Thin-film composite pressure retarded osmosis membranes for sustainable power generation from salinity gradients. Environ. Sci. Technol..

[44] Lonsdale H.K., Riley R.L., Lyons C.R., Carosella D.P. (1971). Transport in Composite Reverse Osmosis Membranes. Membrane Processes in Industry and Biomedicine.

