# Supplementary material for: Role of Nanocomposite Support Stiffness on TFC Membrane Water Permeance
Source: Membranes (Basel). 2018 Nov 18;8(4):111. doi: 10.3390/membranes8040111 (PMC6315447; doi:10.3390/membranes8040111)
Supplement: Supplementary file 1 [file membranes-08-00111-s001.pdf]

membranes

Supplementary Materials

Role of Nanocomposite Support Stiffness on TFC Membrane Water Permeance

Jaime A. Idarraga-Mora [1], Anthony S. Childress [2], Parker S. Friedel [1], David A. Ladner [3], Apparao M. Rao [2] and Scott M. Husson [1,*]

[1] Department of Chemical and Biomolecular Engineering, Clemson University, 127 Earle Hall, Clemson, SC 29634, USA; jidarra@g.clemson.edu (J.A.I.-M.); psfried@g.clemson.edu (P.S.F.)
[2] Department of Physics and Astronomy, and Clemson Nanomaterials Institute, Clemson University, Clemson, SC 29634, USA; childre@g.clemson.edu (A.S.C.); arao@clemson.edu (A.M.R.)
[3] Department of Environmental Engineering and Earth Sciences, Clemson University, 342 Computer Court, Anderson, SC 29625, USA; ladner@clemson.edu
* Correspondence: shusson@clemson.edu; Tel: +1-864-656-4502, Fax: +1-864-656-0784

CNTs Synthesis and Dispersion

HCNTs were grown on quartz substrates via chemical vapor deposition. The quartz pieces were arranged within a 3.8 cm inner diameter quartz tube and placed within a ThermCraft tube furnace. The catalyst solution used during synthesis consisted of 66.7 mg·cm^{-3} ferrocene, 82.2 mg ml^{-1} indium isopropoxide, and 26.4 mg·cm^{-3} tin isopropoxide dissolved in o-xylene. Before injecting the catalyst solution, the main furnace and pre-heating furnace were allowed to equilibrate at 750 °C and 200 °C under a flow of 500 sccm argon and 100 sccm hydrogen. Once the temperatures were stable, acetylene was introduced at 40 sccm and the catalyst solution was injected into the preheating furnace at 1.5 cm^3 hr^{-1}. Reaction times were approximately 30 min, after which the furnaces were allowed to cool under argon flow before samples were removed.

Figure S1 shows the IR transmission spectra of CNTs before and after functionalization. Following treatment, a prominent OH peak is present just below 3500 cm^{-1}, while a peak at ~1700 cm^{-1} is likely to be due to C=O groups. These groups improve the dispersion of CNTs in N-methyl-2-pyrrolididone (NMP).

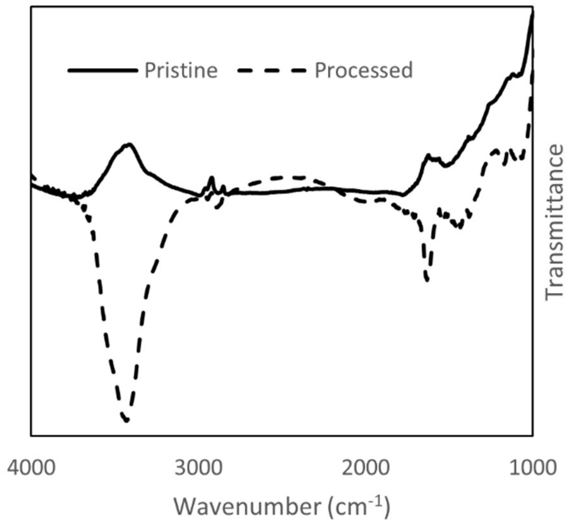

Figure S1. Typical IR spectra of pristine and functionalized CNTs.

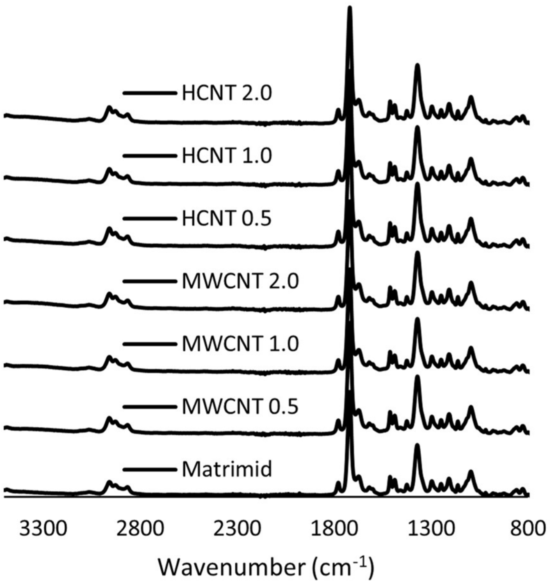

Figure S2. ATR-FTIR spectra of composite supports with different CNT loads.

Derivation of the Model for Predicting Young's modulus of Porous Xomposite < aterials

The assumptions of this model are:

- There is full contact between the polymer and filler
- The efficiency of the contact depends mainly on the aspect ratio of the filler
- Polymeric membrane materials can reach high porosity (>70%)
- Membranes do not swell due to ambient humidity at room temperature

Table S1 provides the nomenclature for model development.

Table S1. Nomenclature used in model.

f = volumetric fraction of filler	W = Width
n = adjustable parameter	ε = strain
A = Area perpendicular to force	η_{Aspect} = Contact Efficiency
E = Young's modulus	σ = tensile stress
F = force applied	ϕ = porosity
G = Shear Stress	ϕ_0 = adjustable porosity parameter
H = Height	Δ = length of deformation

Consider a fully solid composite material like that shown in Figure S3 (left). Now consider the control volume shown in Figure S3 (right). This control volume has a total height defined as 1 unit, which is the addition of the individual heights of each component, which are proportional to the volume fraction of each material in the initial composite. For the heights to be additive based on volume fractions, both components must be in full contact. This is the main assumption used in the Rule of Mixtures [1S].

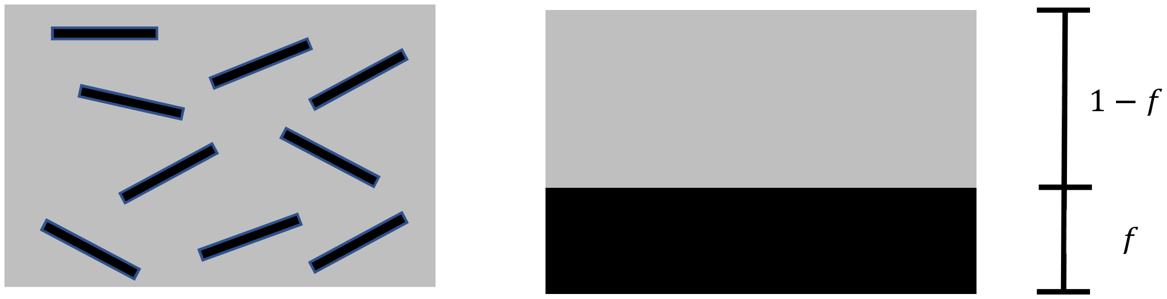

Figure S3. (**Left**) Schematic representation of a fully solid composite material, where the matrix is a polymer and the filler is a carbonaceous material. (**Right**) Control volume describing the interface between the matrix and the filler, where the total height is 1 unit, and the component heights are proportional to their volume fractions.

Consider a force applied to the control volume. This force can be axial (horizontal) or transversal (vertical) loading. Since the fillers in the actual composite will have an orientation distribution, these two loading cases are limiting scenarios, and the real behavior should be somewhere in between, depending on the actual orientation distribution.

In the case of axial (horizontal) loading, we assume that the strain in each component is equal:

$$\varepsilon_{\text{matrix}} = \varepsilon_{\text{filler}} = \varepsilon_{\text{composite}} \tag{S1}$$

Also, by definition we have that:

$$\varepsilon = \frac{\sigma}{E} \tag{S2}$$

Doing a force balance, we can say that:

$$F_{\text{matrix}} + F_{\text{filler}} = F_{\text{composite}} \tag{S3}$$

Now, taking into account the definition of stress:

$$\sigma = \frac{F}{A} = \frac{F}{W \times H} = E\varepsilon \tag{S4}$$

We substitute this into the force balance, obtaining:

$$(E\varepsilon WH)_{\text{matrix}} + (E\varepsilon WH)_{\text{filler}} = (E\varepsilon WH)_{\text{composite}} \tag{S5}$$

We know that the width and strain are equal for each element. Additionally, the total height of the control volume (composite) is 1 unit, and the height of each component is proportional to its volume fraction. Finally, we simplify:

$$(1-f)E_{\text{matrix}} + fE_{\text{filler}} = E_{\text{composite}} \tag{S6}$$

In the case of transverse (vertical) loading, we assume that the force applied in each component is equal; therefore the stress is equal,

$$\sigma_{\text{matrix}} = \sigma_{\text{filler}} = \sigma_{\text{composite}} \tag{S7}$$

By definition, the strain is the deformation over the original length:

$$\varepsilon_{\text{matrix}} = \frac{\Delta_{\text{matrix}}}{1-f} \tag{S8}$$

$$\varepsilon_{\text{filler}} = \frac{\Delta_{\text{filler}}}{f} \tag{S9}$$

$$\varepsilon_{\text{composite}} = \frac{\Delta_{\text{matrix}} + \Delta_{\text{filler}}}{(1-f)+f} \tag{S10}$$

Solving S8 and S9 for the lengths of deformation and substituting these into S10, and applying the definition of stress (S4) for the composite and each of the individual components yields:

$$\varepsilon_{\text{composite}} = \frac{\sigma_{\text{composite}}}{E_{\text{composite}}} = (1-f)\varepsilon_{\text{matrix}} + f\varepsilon_{\text{filler}} \quad \text{(S11)}$$

$$\frac{\sigma_{\text{composite}}}{E_{\text{composite}}} = (1-f)\frac{\sigma_{\text{matrix}}}{E_{\text{matrix}}} + f\frac{\sigma_{\text{filler}}}{E_{\text{filler}}} \quad \text{(S12)}$$

Because the stress is equal in each component, this equation finally simplifies to:

$$E_{\text{composite}} = \left(\frac{1-f}{E_{\text{matrix}}} + \frac{f}{E_{\text{filler}}}\right)^{-1} \quad \text{(S13)}$$

These formulas represent the Rule of Mixtures in Material Science [1S]. Cox proposed an efficiency factor that is associated with the aspect ratio of the filler, to correct the calculation of the modulus of the composite [2S]. This factor is determined from Equations S14 to S17:

$$\eta_{Aspect} = 1 - \frac{tanh(\alpha\beta)}{\alpha\beta} \quad \text{(S14)}$$

$$\beta = \sqrt{\frac{2G_{\text{matrix}}}{E_{\text{filler}}\ln\delta}} \quad \text{(S15)}$$

$$\alpha = \text{length/diameter} \quad \text{(S16)}$$

$$\delta = \sqrt{\frac{2\pi}{\sqrt{3}\,f}} \quad \text{(S17)}$$

The corrected modulus equations for axial and transverse loading are:

$$E_{\text{composite}} = (1-f)E_{\text{matrix}} + \eta_{Aspect} f E_{\text{filler}} \quad \text{(S18)}$$

$$E_{\text{composite}} = \left(\frac{1-f}{E_{\text{matrix}}} + \frac{f}{\eta_{Aspect}E_{\text{filler}}}\right)^{-1} \quad \text{(S19)}$$

Equations S18 and S19 were developed for fully solid composites. To extend the applicability to porous membranes, it is necessary to account for the effect of voids in the body of the composite. Roberts and Garboczi proposed an equation to estimate the mechanical properties of porous ceramic materials [3S].

$$\frac{E}{E_{\text{solid}}} = \left(1 - \frac{\phi}{\phi_0}\right)^n \quad \text{(S20)}$$

The adjustable porosity parameter can be seen as the highest porosity value that sustains a self-supporting structure. It is a limiting value. In the case of polymeric membranes, which can be highly porous, this value approaches 1. With this assumption:

$$\frac{E}{E_{\text{solid}}} = (1-\phi)^n \quad \text{(S21)}$$

This equation only has one adjustable parameter, n. Values of n have been reported for ceramics, and they usually fall in the range from 1.8 to 2.4 [3A]. Substituting Equations S18 and S19 for E_{solid} in Equation S21 yields formulas for the upper-bound limit and the lower-bound limit for Young's Modulus of polymeric porous films (e.g., membranes):

$$E_{\text{membrane}}^{\text{upper-bound}} = \left((1-f)E_{\text{matrix}} + \eta_{Aspect} f E_{\text{filler}}\right)(1-\phi)^n \quad \text{(S22)}$$

$$E_{\text{membrane}}^{\text{lower-bound}} = \left(\frac{1-f}{E_{\text{matrix}}} + \frac{f}{\eta_{Aspect} E_{\text{filler}}}\right)^{-1} (1-\phi)^n \quad \text{(S23)}$$

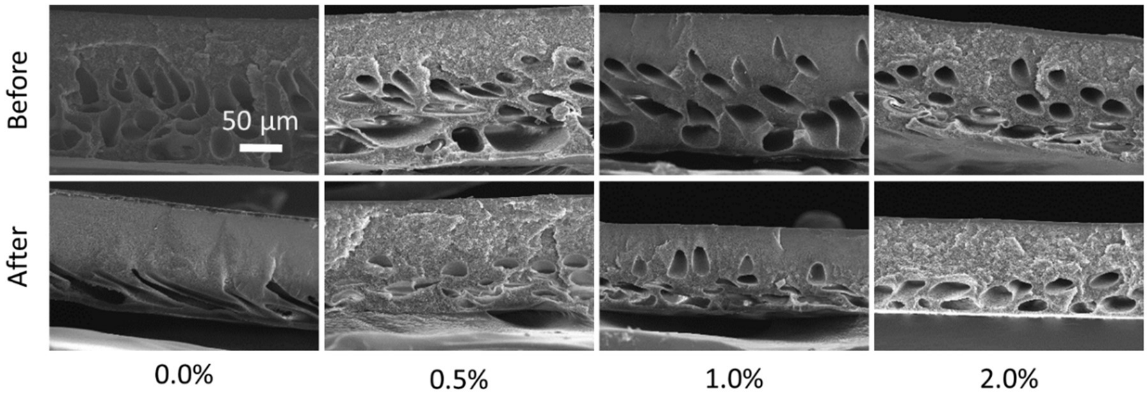

Figure S4. Typical cross-sectional SEM images of TFC membranes using composite supports before and after two-stage pressure stepping water flux measurements.

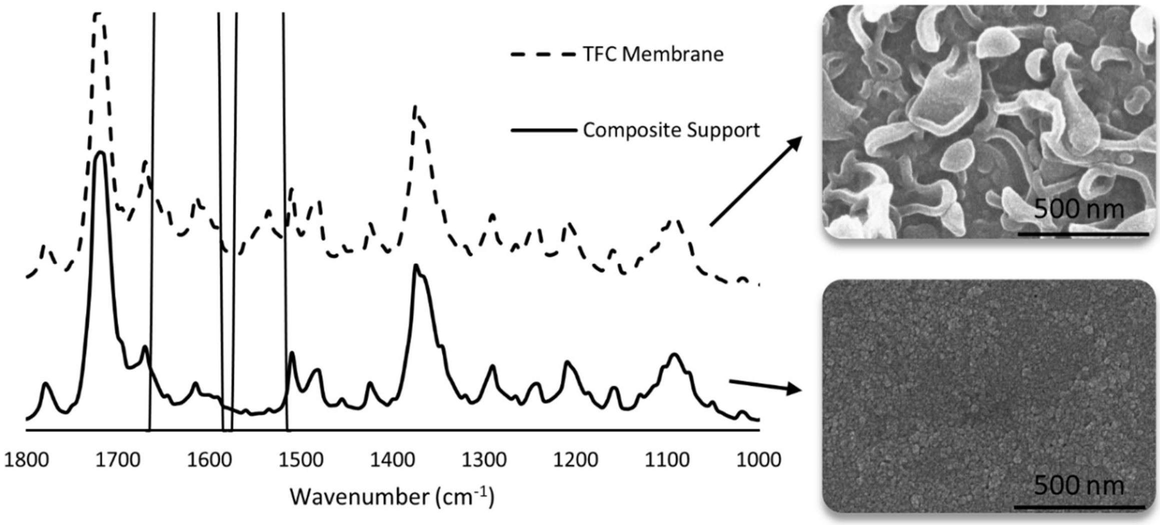

Figure S5. (**Left**) ATR-FTIR spectra comparison of TFC membranes and composite support (polyamide formation). (**Right**) SEM imaging of the top surface of the TFC membrane.

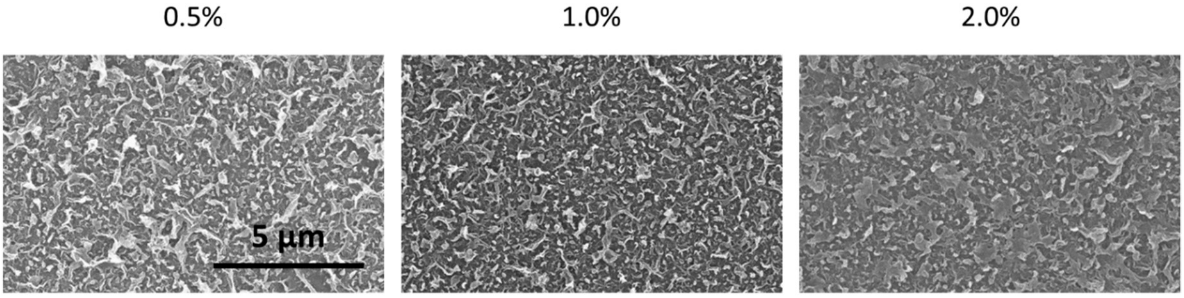

Figure S6. SEM images of the top surface of the TFC membranes after interfacial polymerization on nanocomposite upports with different CNT loading.

Table S2. Statistical analysis of the effect of CNT loading of nanocomposite supports in the water permeance (*A*) of the respective TFC membranes.

CNT load	A (LMH/bar)	s	n	t observed	t critical	p-value
0.0%	0.65	0.07	3	0.00	2.78	1.00
0.5%	0.66	0.28	3	−0.11	4.30	0.92
1.0%	0.70	0.23	3	−0.37	4.30	0.74
2.0%	0.66	0.12	3	1.03	3.18	0.38

Table S3. Statistical analysis of the effect of CNT loading of nanocomposite supports in the NaCl rejection of the respective TFC membranes.

CNT load	Salt Rejection	s	n	t observed	t critical	p-value
0.0%	0.91	0.05	3	0.00	2.78	1.00
0.5%	0.87	0.04	3	1.15	3.18	0.34
1.0%	0.92	0.03	3	−0.07	3.18	0.95
2.0%	0.78	0.05	3	3.17	3.18	0.05

References for Supplementary Materials

1S. Askeland, D.R.; Wright, W.J. *Essentials of Materials Science & Engineering*, 3rd ed.; Cengage Learning: Stamford, CT, USA, 2013.

2S. Cox, H.L. The elasticity and strength of paper and other fibrous materials. *Br. J. Appl. Phys.* **1952**, *3*, 72–79.

3S. Roberts, A.P.; Garboczi, E.J. Elastic properties of model porous ceramics. *J. Am. Ceram. Soc.* **2000**, 83, 3041–3048.

© 2018 by the authors. Submitted for possible open access publication under the terms and conditions of the Creative Commons Attribution (CC BY) license (http://creativecommons.org/licenses/by/4.0/).
